# Pathways to experienced coercion during psychiatric admission: a network analysis

**DOI:** 10.1186/s12888-024-05968-w

**Published:** 2024-08-02

**Authors:** Benedetta Silva, Stéphane Morandi, Mizue Bachelard, Charles Bonsack, Philippe Golay

**Affiliations:** 1 Community Psychiatry Service, Department of Psychiatry, Lausanne University Hospital and University of Lausanne, Lausanne, Switzerland; 2 Cantonal Medical Office, General Directorate for Health, Canton of Vaud, Department of Health and Social Action, Lausanne, Switzerland; 3 General Psychiatry Service, Department of Psychiatry, Lausanne University Hospital and University of Lausanne, Lausanne, Switzerland; 4 Institute of Psychology, Faculty of Social and Political Sciences, University of Lausanne, Lausanne, Switzerland

**Keywords:** Coercion, Experience, Perceived coercion, Psychiatric hospitalisation, Treatment, Network analysis

## Abstract

**Background:**

In mental health care, experienced coercion, also known as perceived coercion, is defined as the patient’s subjective experience of being submitted to coercion. Besides formal coercion, many other factors have been identified as potentially affecting the experience of being coerced. This study aimed to explore the interplay between these factors and to provide new insights into how they lead to experienced coercion.

**Methods:**

Cross-sectional network analysis was performed on data collected from 225 patients admitted to six psychiatric hospitals. Thirteen variables were selected and included in the analyses. A Gaussian Graphical Model (GGM) using Spearman’s rank-correlation method and EBICglasso regularisation was estimated. Centrality indices of *strength* and *expected influence* were computed. To evaluate the robustness of the estimated parameters, both edge-weight accuracy and centrality stability were investigated.

**Results:**

The estimated network was densely connected. Formal coercion was only weakly associated with both experienced coercion at admission and during hospital stay. Experienced coercion at admission was most strongly associated with the patients’ perceived level of implication in the decision-making process. Experienced humiliation and coercion during hospital stay, the most central node in the network, was found to be most strongly related to the interpersonal separation that patients perceived from staff, the level of coercion perceived upon admission and their satisfaction with the decision taken and the level of information received.

**Conclusions:**

Reducing formal coercion may not be sufficient to effectively reduce patients’ feeling of being coerced. Different factors seemed indeed to come into play and affect experienced coercion at different stages of the hospitalisation process. Interventions aimed at reducing experienced coercion and its negative effects should take these stage-specific elements into account and propose tailored strategies to address them.

**Supplementary Information:**

The online version contains supplementary material available at 10.1186/s12888-024-05968-w.

## Introduction

In mental health care, experienced coercion is defined as the patient’s subjective experience, consideration and feeling of being submitted to coercion [1]. Because of its demonstrated negative effects, this phenomenon, also known as perceived coercion, has been extensively studied during the last decades. Indeed, evidence have shown that feeling coerced into treatment negatively affects the therapeutic relationship with staff [2, 3], decreases patients’ collaboration [4] and satisfaction with treatment [5–8], and increases the risk of suicide attempts after discharge [9].

Patients may feel coerced into treatment because of an “objective coercion” [10]. Indeed, in psychiatry, several coercive measures can be legally enforced on patients to compel them to undergo treatment. The specific circumstances and criteria under which these procedures may be applied vary from country to country according to the local legislation. However, the most widespread formal coercive measures used in psychiatry include involuntary hospitalisation, involuntary outpatient treatment, also known as community treatment orders, seclusion, restraint and forced medication [11].

Besides objective coercion, a large number of other factors have been identified as potentially affecting patients’ feeling of being coerced during psychiatric hospitalisation. Indeed, evidence suggests that patients may perceive high level of coercion regardless of their admission status or the coercive measures to which they have been submitted [12–14]. Several forms of informal coercion, such as persuasion, leverage and threat [10, 15, 16], but also unwelcome predictions, advice, offers or repeated requests [1, 10, 17, 18], can be experienced by patients as coercive. These more subtle forms of coercion may be applied in place of or in addition to formal coercion by both professionals and relatives to promote patient adherence to treatment [19–21]. Hospital practices and rules, such as locked doors and restrictions on visits or contact with others, as well as environmental and architectural elements, can also increase patients’ feeling of being coerced [11, 22, 23].

Finally, patients’ perceived coercion has been found to be strongly affected by the quality of the interaction with professionals [24, 25] and the level of *procedural justice* perceived [26–30]. *Procedural justice* refers to the patient perception that in the decision-making process professionals treated them fairly (fairness), listened to them (voice), took their preferences into account (validation), respected them (respect), genuinely cared about them (motivation) and sufficiently informed them (information) [31].

Despite the high number of quantitative and qualitative studies trying to identify which factors most affected patients’ feeling of being coerced, to date none has examined how they interacted with each other and which ones played a key role in this process. This study aimed to fill this gap by exploring the interplay between the different factors affecting patients’ feeling of being coerced during hospitalisation, and to provide new insights into how they may lead to experienced coercion. Factors were selected according to the results of a previous literature review [32] and a qualitative study [33]. These studies have shown that, from the patients’ perspective, contextual and relational factors, such as level of information received, involvement in the decision-making process and staff attitudes, as well as satisfaction with hospital treatment, were indeed main determinants of the experience. Therefore, by taking these factors into account and including them in a network model, the present study sought to go a step further and identify which ones played a key role in the process. Network analysis is a sophisticated analytical approach that allows to explore and graphically represent the structure of relationships in multivariate data without relying on an a priori model [34]. In network models, variables are represented as *nodes* and their association as *edges*. The strength of the relationship between nodes (*edge weight*) can be statistically estimated from the data and inference methods can be used to assess which nodes play the most important role in the network (*nodes centrality*) [35]. Although recent years have seen an important growth of network studies in psychology and psychiatry research, to the best of our knowledge, no study has so far used these models to investigate experienced coercion. Network models offer powerful tools to clarify the complex pathways that lead to experienced coercion. Identifying the factors that play a central role in this process is of paramount importance in order to develop tailored strategies to tackle them.

## Methods

### Participants

Participants were recruited between March 2020 and June 2022 in six psychiatric hospitals of three French-speaking Swiss cantons. Eligible participants had to be aged between 18 and 65 years, be sufficiently proficient in French and be able to provide a formal consent. Only patients hospitalized between 7 and 15 days were assessed and included in the study. This inclusion criterion allowed to reduce sample variability in terms of duration of hospitalisation and minimize the risk of memory bias in case of long-lasting stay. People suffering from dementia (F0) or intellectual disability (F7) were excluded from the study. Eligible participants were approached directly on site by a trained research assistant, independent of the hospital team. After receiving detailed information about the study, those who agreed to participate were asked to sign a written informed consent. All assessments were administered during one session immediately following the inclusion in the study.

The study was carried out in accordance with the Human Research Ethics Committee of the Canton Vaud and the Declaration of Helsinki. Approval was granted by the Human Research Ethics Committee of the Canton Vaud, Switzerland (protocol #2016–00768).

### Measures

The measures included in the study were selected based on the results of a previous literature review [32] and a qualitative study [33] aimed at exploring patients’ perspectives on experienced coercion and the factors most affecting it during psychiatric hospitalisation.

#### Experienced coercion

Participants’ experienced coercion at admission was measured using the French version of the MacArthur Admission Experience Survey (AES) short form [36]. Including 16 true-false items, this questionnaire provides three sub-scores (Perceived Coercion, Negative Pressures and Voice) and a total score. Higher scores indicate higher perceived coercion at admission. For the purpose of this study, only the AES total score was included in the analysis.

Since the AES only refers to the hospital admission process, it is not suitable to capture the impact of other coercive measures which may occur during the hospitalisation [37]. For this purpose, the Coercion Experience Scale [CES; 38] was included in the study. The CES measures the extent to which patients feel that their human rights, such as human dignity, autonomy, freedom of movement, physical inviolability and contact with staff, have been violated during the coercive measure and how this has negatively affected them. Moreover, this instrument takes into account the psychological impact of a wide range of interindividual and contextual stressors which may occur when experiencing coercion during the hospitalisation. The French version of the CES includes 31 Likert-type items and allows to compute five sub-scores: Humiliation/coercion score (CES-H); Physical adverse effects score (CES-PAE); Interpersonal separation score (CES-IS); Negative environmental influences score (CES-NEI) and Fear score (CES-F). In the instructions, coercive measures were defined as: confinement, restriction of freedom of movement or of contact with family and friends, seclusion, restraint or pharmacological contention. All five sub-scores were included in the analysis.

#### Perceived fairness and effectiveness

Patients’ perceived fairness of treatment pressures was assessed using the Index of fairness [39]. Participants were asked to answer the following items “Overall, the pressures or things people have done to try to get me into treatment or stay in treatment (1) Were done by people who tried to be fair to me (2) Were done for my own good (3) Were not done out of real concern for me (reverse coded) (4) Didn’t make me feel respected as a person (reverse coded)”.

Patients’ perceived effectiveness of treatment pressures was assessed using the Index of effectiveness [39]. Participants were asked to answer the following item: “Overall, the pressures or things people have done to try to get me into treatment or stay in treatment (1) Made me more likely to keep appointments and take my medications (2) Helped me get well and stay well (3) Helped me gain more control over my life (4) Should be done again in the future”.

All items were rated on a Likert scale ranging between 1 (strongly disagree) to 5 (strongly disagree). A total score for each index was computed summing the answers to the four items.

#### Formal coercion

Formal coercion was defined as the number of coercive measures undergone by each participant during the first 7 days of their hospital stay. Coercive measures included involuntary hospitalisation, involuntary hospitalisation following voluntary admission, seclusion, restraint, forced medication and locked doors. Information on formal coercive measures were directly extracted from the participants’ medical records and transmitted to the research assistant by the hospital staff.

#### Informal coercion

Following Burns et al. (2011), informal coercion was assessed using a 4-item instrument adapted from Monahan et al. (2005). Participants were asked to report experiences of leverage in four social welfare domains: finance, housing, criminal justice and child custody. These items represent rather severe forms of informal coercion corresponding to inducements and threats in the Szmukler & Applebaum’s model [10]. Answers were dichotomous (yes/no). For the purpose of this study, the number of positive responses provided by each participant was computed and included in the analysis.

#### Implication in decision-making

The patient version of the Clinical Decision-making Involvement and Satisfaction scale [CDIS-P; 40] was used to assess participants’ involvement in the decision of being hospitalized. This instrument is composed of two sub-scales: Satisfaction and Implication.

The Satisfaction sub-scale (CDIS-S) aims to measure patients’ satisfaction with the decision taken and the level of information received in the decision-making process. Based on the answers to six five-point Likert-type items, a mean total score was computed ranging from 1 (low satisfaction) to 5.

The Implication sub-scale (CDIS-I) aims to measure patients’ level of perceived involvement in the decision process. Participants were asked to rate their level of involvement choosing among five categories, ranging between 1 (active) and 5 (passive). For the purpose of this study, the CDIS-I score was rescaled (0–4) and reversed in order for higher scores to indicate more active involvement.

#### Satisfaction with hospitalisation

Satisfaction with hospitalisation was assessed using a structured questionnaire developed by the Swiss National Association for Quality development in hospitals and clinics [ANQ, 41]. This instrument includes six items measuring patient satisfaction with quality of treatment, information and communication, medication, implication, and discharge preparation on a five-point Likert scale. The total score was computed and included in the analysis, with higher score indicating higher global satisfaction with the hospitalisation.

### Statistical analysis

Cross-sectional network analysis was performed on completely observed data (*N* = 225). Thirteen variables were selected and included in the analyses. Due to the nature of the data, Pairwise Markov Random Field model was estimated [35]. PMRF is a class of undirected network models in which nodes represent variables and edges represent the strength of the conditional dependence between two nodes after controlling for all other variables in the network [42]. The best PMRF model to use must be selected based on the type of available data. Our dataset included non-normal continuous data (see Table 1s in Supplementary File 1). Only one variable was ordered categorical (CDIS-I). Following Blanken et al. (2022), all variables were treated as continuous and regularized Gaussian Graphical Model (GGM) was estimated using Spearman’s rank-correlation method. GLASSO (Graphical LASSO) regularisation was applied to limit the number of spurious edges in the network [44]. The optimal tuning parameter (λ) defining the amount of penalization applied was selected by minimizing the Extended Bayesian Information Criterion [EBIC; 43]. The EBIC hyperparameter (*y*), which controls the amount of extra penalization applied to model complexity [43], was set to the default value of 0.5. The estimated network was represented using the Fruchterman and Reingold algorithm [45], which displays nodes with higher centrality in the centre and those more disconnected in the periphery [46]. The thickness of the edge indicates its weight (thicker edges for stronger partial correlations), while the colour designates its sign (blue for positive partial correlations and red for negative partial correlations). No specific minimum/maximum cut values were used.

In order to quantify the importance of each node in the network, centrality indices of *strength* and *expected influence* were computed. Node’s *strength* quantify how well a node is connected to the others based on the sum of its absolute edge-weights [35]. *Expected influence* (EI) identify how influential is a node based on the sum of its positive and negative edges [47]. Centrality plots were created to represent raw and standardized scores for both indices [48].

Finally, to evaluate the robustness of the estimated parameters, both edge-weight accuracy and centrality stability were investigated [35]. Edge-weight accuracy was tested through non-parametric bootstrapping with 1000 bootstrap samples. Bootstrapped difference tests between all pairs of edge-weights were also performed to show if some edges differed consistently from others [49]. Centrality stability allows to estimate the stability of the order of centrality indices [35]. It was tested through case-drop bootstrapping with 1000 bootstrap samples. Correlation stability coefficient (CS(cor = 0.7)) was computed for both centrality indices [35]. In order for centrality indices to be interpretable, CS-coefficient should preferably be above 0.5 and in any case not be lower than 0.25 [49]. Finally, bootstrapped difference tests of centrality indices were run for both, nodes’ strength and expected influence.

Descriptive analyses were conducted using IBM SPSS 27. Network analysis was performed using R [Version 4.3.0; 50]. The bootnet [Version 1.5.1; 35] and qgraph packages [Version 1.9.5; 51] were used to estimate and visualize network structure, centrality indices and their accuracy and stability.

## Results

### Sample characteristics

Globally, 225 patients with no missing data out of 230 were included in this study. The study sample consisted of 120 (53.3%) women. Participants were aged between 18 and 64 years (M = 39.2; SD = 13.7). Most were single (*n* = 133; 59.1%) and of Swiss nationality (*n* = 165; 73.3%). Seventy-one (31.6%) were formally involuntarily hospitalized while 154 (68.4%) were voluntarily admitted. Descriptive statistics for sample socio-demographic and clinical characteristics are presented in Table 2s of the Supplementary Material (Supplementary File 1).

### Network analysis

**Fig. 1:**
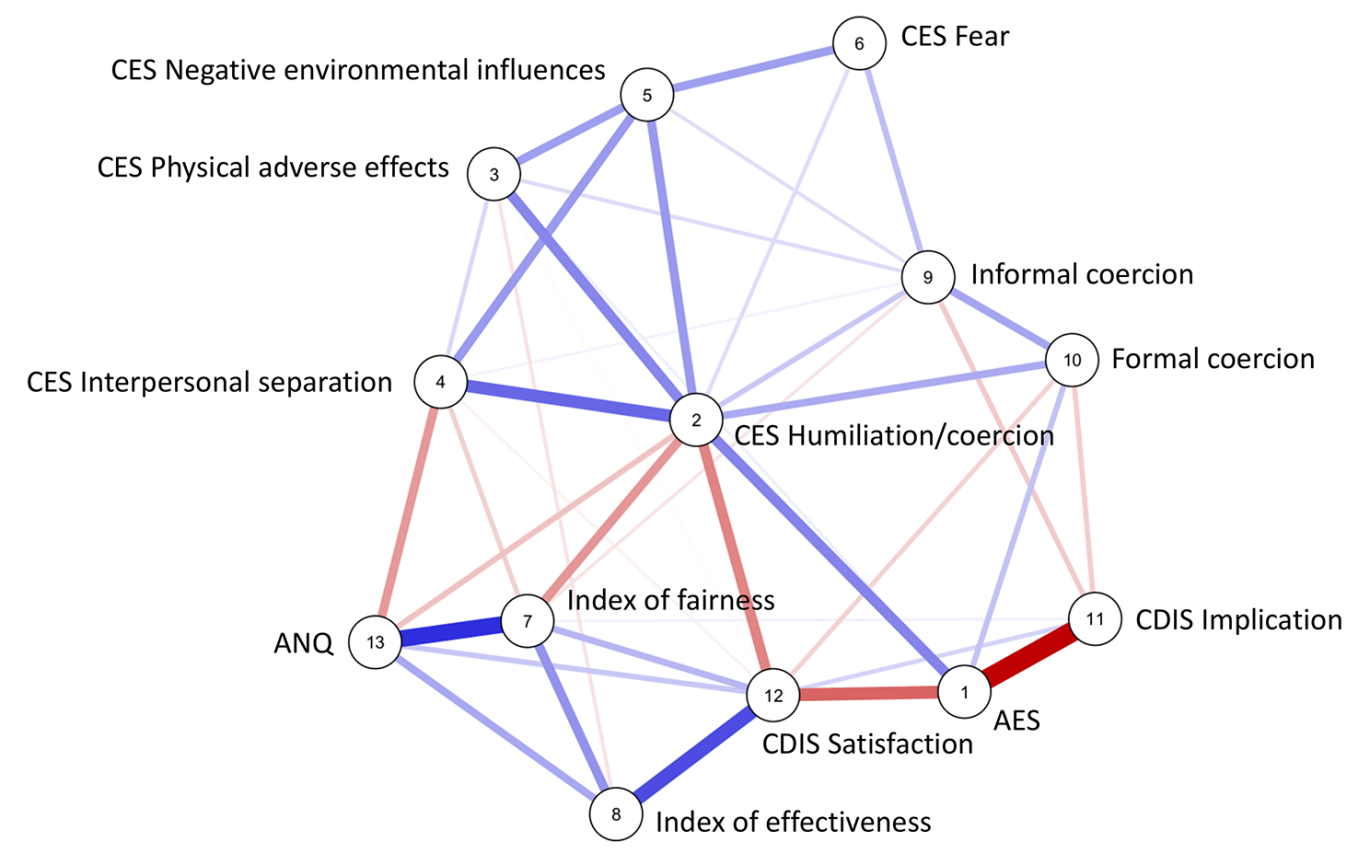
Estimated Gaussian Graphical Model (GGM) of experienced coercion.The thickness of the edge reflects the magnitude of the association. Blue lines represent positive partial correlations, whereas red lines represent negative partial correlations. Note. AES MacArthur Admission Experience Survey; CES Coercion Experience Scale; CDIS Clinical Decision-making Involvement and Satisfaction scale; ANQ Satisfaction questionnaire developed by the Swiss National Association for Quality development in hospitals and clinics

The estimated network is visualised in Fig. 1. Overall, 40 non-zero-edges out of 78 potential connections were contained in the network, resulting in a density of 0.513. As expected, both positive and negative interconnections were found between nodes. Negative edges ranged between − 0.005 (CES Physical adverse effects - CDIS Satisfaction) and − 0.394 (AES - CDIS Implication). Positive edges ranged between 0.018 (CES Interpersonal separation - Informal coercion) and 0.323 (Index of fairness - ANQ). The network mean weight was 0.02. The whole weighted adjacency matrix is provided in Table 3s of the Supplementary Material (Supplementary File 1).

**Fig. 2:**
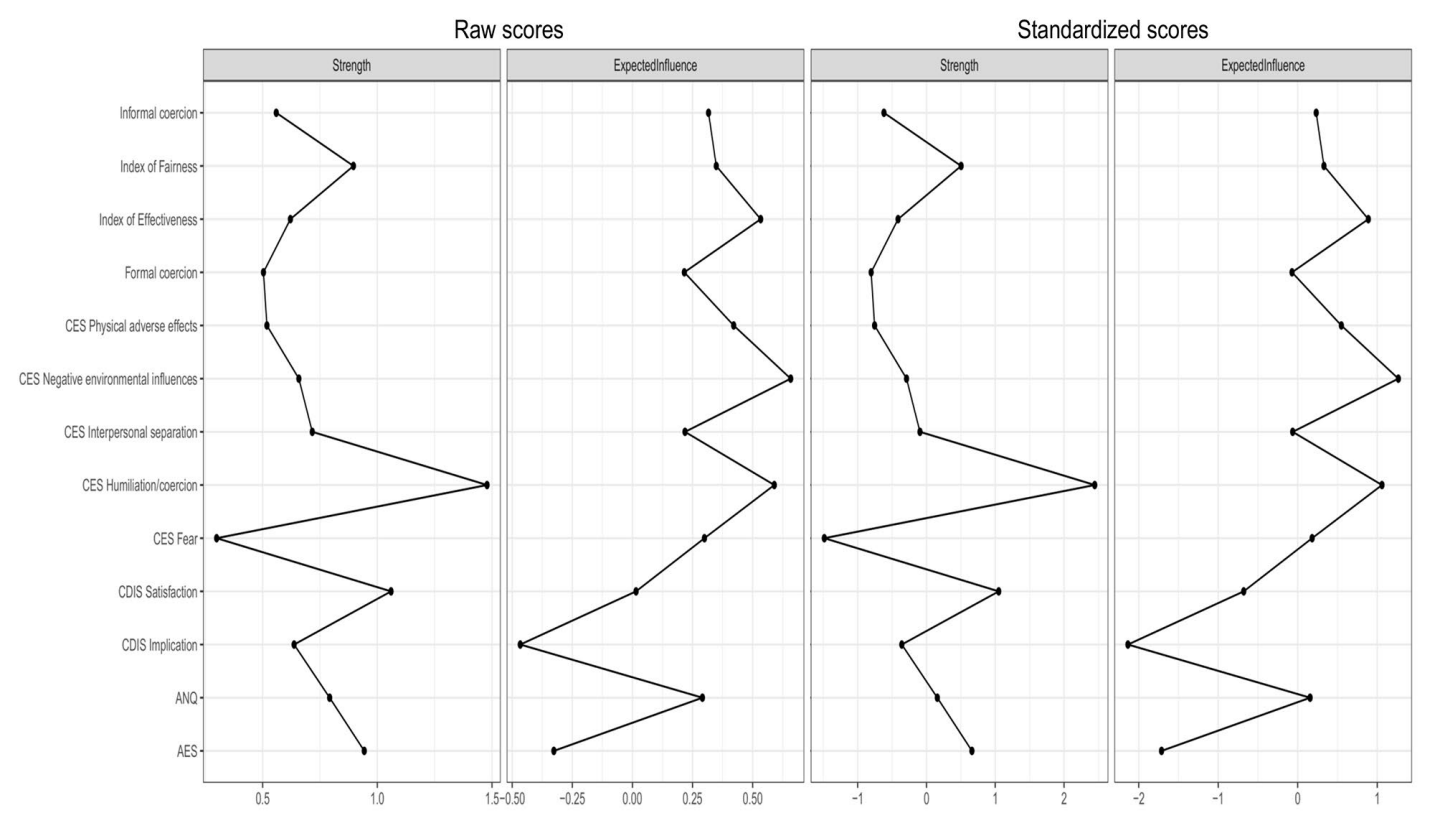
Centrality plots depicting raw and standardized estimates of strength and expected influence for each node in the network. Note. AES MacArthur Admission Experience Survey; CES Coercion Experience Scale; CDIS Clinical Decision-making Involvement and Satisfaction scale; ANQ Satisfaction questionnaire developed by the Swiss National Association for Quality development in hospitals and clinics

Centrality indices as raw and standardized scores are represented in Fig. 2. Both raw and standardized scores are also reported in Table 4s of the Supplementary Material (Supplementary File 1).

The CES Humiliation/coercion node showed the greatest *strength* in the network, followed by CDIS Satisfaction. CES Fear was, on the contrary, the weakest node in the network. The nodes with the highest positive *expected influence* in the network were the CES Negative environmental influences, the CES Humiliation/coercion, and the Index of effectiveness. On the negative side, the CDIS Implication and the AES showed the strongest expected influence. These results were confirmed for both, raw and standardized scores. However, for the expected influence the order of importance of the nodes was slightly different depending on the type of score taken into consideration.

After controlling for all other variables in the network, the CES Humiliation/coercion showed positive associations with the CES Interpersonal separation (0.235), the AES (0.191), the CES Physical adverse effects (0.187) and the CES Negative environmental influences (0.158). Positive but weaker links were also found with formal coercion (0.130), informal coercion (0.083) and CES Fear (0.052), while negative edges emerged with the CDIS Satisfaction (-0.191), the Index of fairness (-0.162) and the ANQ (-0.093).

In addition to the CES Humiliation/coercion (0.191), the AES was inversely correlated with the CDIS Implication (-0.394) and the CDIS Satisfaction (-0.240). A weak positive edge was also found with formal coercion (0.093) and CES Physical adverse effects (0.024), while no link emerged with informal coercion.

Finally, no association was identified between the ANQ and formal coercion, informal coercion, or AES score. The ANQ was instead found to be positively correlated with the Index of fairness (0.323), the Index of effectiveness (0.133) and the CDIS Satisfaction (0.085), and inversely correlated with the CES Interpersonal separation (-0.158) and the CES Humiliation/coercion (-0.93).

### Network accuracy and stability

The edge-weight bootstrap revealed moderate CIs around edge-weights, indicating that the estimation was adequate (Fig. 1s in Supplementary File 1). The edge-weight difference test confirmed that the network was accurately estimated. The strongest edge in the network (AES – CDIS Implication) was significantly stronger than any other. The other strongest edges differed significantly from some of the edges with lower strength (Fig. 2s in Supplementary File 1). The order of strength centrality and expected influence estimates was stable, with a centrality stability coefficient of 0.51 for both indices (Fig. 3s in Supplementary File 1). The strength centrality difference test showed a significant difference between nodes with the highest strength and nodes with the lowest strength (Fig. 4s in Supplementary File 1). The difference test for expected influence was in line with the standardized z-score results of the original sample network. Indeed, nodes with the highest positive expected influence differed significantly from nodes with the highest negative expected influence but not from nodes with lower positive expected influence. On the contrary, nodes with the highest negative expected influence differed significantly from all other nodes except each other (Fig. 5s in Supplementary File 1).

## Discussion

This study aimed to explore the interplay between different factors affecting patients’ feeling of being coerced, in order to provide new insights into how they interact and which of them play a key role in the process leading to experienced coercion. To this purpose network analyses were used.

The estimated network was densely connected, confirming the complex relationship between the included factors and the subjective experience of coercion. The network being undirected, no causal inferences can be drawn from the results. However, comparing them with what previously observed in the scientific literature, allow us to suggest some interpretations and propose important implications for practice and research.

In the estimated network, formal coercion was only weakly associated with experienced coercion both at admission and during hospital stay. This confirms what already pointed out in the literature about the need to move research on experienced coercion beyond formal coercion [22, 23, 52]. Several studies have indeed demonstrated that patients may perceive high level of coercion even when no coercive measures are enforced on them, and vice versa [14, 53–56]. Our study went a step further showing which were the other factors more strongly associated with experienced coercion than formal coercion itself.

Experienced humiliation and coercion during hospital stay was the most strongly connected node in the network. Due to the nature of the analysed data, it is not possible to conclude whether this node was the most central because it was the most affected or because it was the most influential in the network. However, we can point out that, during hospital stay, experienced humiliation/coercion was most strongly associated with the degree of interpersonal separation experienced by patients towards staff members. Several qualitative and quantitative studies have previously highlighted the key role of the relationship with professional in shaping patients’ experience of coercion during both voluntary and involuntary hospital admission [25, 32]. Perceiving closeness and empathy from staff has been reported to decrease patients’ feeling of being coerced and disrespected [57–68], making even formal coercion more acceptable [69, 70]. Several contextual factors, such as discontinuity or lack of time with professional, hospital practices and rules or its environmental and architectural elements, may impact patients’ relationship with staff and feeling of being coerced [11, 22, 23, 71]. In our network, experienced negative environmental influences were found to partially correlate with both perceived interpersonal separation and humiliation/coercion during hospital stay. Drawing on these results and on the underpinning literature, we posit that reducing patients’ perception of interpersonal separation towards staff, also by improving the environmental conditions of the hospital, could effectively reduce their feelings of humiliation and coercion during hospitalisation.

The second factor most strongly associated with the level of coercion experienced by patients during hospital stay was the level of coercion they have perceived at admission. Experienced coercion at admission was both directly and indirectly linked to experienced humiliation/coercion during hospital stay. Indeed, its effect appear to be partially mediated and decreased by the level of patient satisfaction with the decision taken and the information received. Several previous studies have supported the relevance of the degree of information received by patients and of their involvement in the decision-making process during hospital admission [72–75]. In our network, while satisfaction with the decision taken/level of information received correlated with experienced coercion both at admission and during hospital stay, only the first one was linked to the level of implication in the decision-making process. Likewise, the strong association between perceived coercion and perceived fairness reported in the literature [26–28, 56, 76–78] was only confirmed for experienced humiliation/coercion during hospital stay but not upon admission. Different factors thus seemed to come into play and affect experienced coercion at different stages of the hospitalisation process.

Upon admission, patients mainly need to be actively involved in the decision-making process in order not to feel coerced into hospital admission. However, at a later stage, having positive contact with the staff, being properly informed and perceiving that the decisions taken, even if against their will, were indeed useful and fair, become the most important factors.

Reducing patients’ feeling of being coerced can in return improve their global satisfaction with care [5–7]. In fact, our study has shown that experienced humiliation/coercion during hospital admission was linked to satisfaction with the hospitalization both directly and indirectly through perceived fairness. The strong association revealed between perceived fairness and satisfaction with hospitalization, the second strongest edge of the network, is in line with what observed in a previous study [79]. Building on these results, we can make the hypothesis that improving patients’ perceived fairness could increase their satisfaction with hospitalization, even when this has been experienced as coercive.

Finally, informal coercion showed no connection with experienced coercion at admission and a very weak one with experienced humiliation/coercion during hospital stay. This result could be partially explained by the type of instrument used to measure informal coercion. The four items included in the questionnaire represent only severe forms of treatment pressures, such as inducement and threats, and refer to four specific domains of social welfare (finance, housing, criminal justice and child custody). These severe forms of informal coercion were less frequently used in our sample. In the network, informal coercion, formal coercion and experienced humiliation/coercion during hospital stay were actually all interconnected, leaving room for the hypothesis of a mediating effect of formal coercion. In order to test this hypothesis and improve our understanding of the role of informal coercion in the process of experienced coercion, we need to develop better instruments to measure informal coercion, including a broader range of treatment pressures.

### Study strengths and limitations

The main strength of this study is the use of a sophisticated novel analytical approach to investigate the complex interplay between factors leading to experienced coercion. To our knowledge, this is the first study applying this innovative approach to this research field. Proposing a shift from a dichotomous vision of coercion to a multidimensional one, network analyses could help to promote the development of more effective integrated interventions to reduce its impact on patients.

Some limitations should be pointed out. First, network models are exploratory tools allowing to generate hypothesis [80]. Indeed, as already mentioned, the cross-sectional nature of the data does not allow us to determine the direction of the effects and thus to conclude about their causality. Further research should be performed to establish the validity of our results and explore the direction of the identified associations using longitudinal data. Second, in Pairwise Markov Random Fields models there is a risk of low specificity when the sample size is lower than 300 [43]. To minimize this problem, we used the GLASSO regularisation with EBIC model selection. This model selection algorithm has shown high specificity, especially with low sample sizes, but varying sensitivity [81]. Third, although several important variables were measured and included in the network, other potentially important factors could be missing. Failing to incorporate important factors in a network could lead to misinterpret its structure [82]. Fourth, a topological overlap could exist between the CES sub-scales. Indeed, the CES French version validation study showed that the five factors were substantially correlated [38]. However, the internal validity analysis confirmed that the source of correlation was not unitary and therefore the computation of a total score was not appropriate. The best factor structure of the French CES included five factors. Moreover, observing the estimated network, the five sub-scales showed differential associations with other nodes [82]. These elements led the authors to the conclusion that the five sub-scales should not be collapsed into one single node and that the risk of a topological overlap could be reasonably excluded.

## Conclusions

This study provides important information for all stakeholders called to develop interventions which aim to reduce inpatients’ feeling of coercion and its negative effects. The complexity of the estimated network confirmed the limitation of a dichotomous model of coercion, which opposes coercion with non-coercion. Experienced coercion is a multidimensional phenomenon, affected by several contextual and relational factors even more than by legal coercive measures. Reducing formal coercion without tacking these factors into account may therefore not be sufficient to effectively reduce patients’ feeling of being coerced. Different factors seemed to come into play and affect experienced coercion at different stages of the hospitalisation process. Increasing the implication of patients in decision-making could lessen experienced coercion at admission, even when under formal coercion. Instead, building a feeling of greater closeness between patients and staff, enhancing patients’ satisfaction with the degree of information received and decision made, and increasing their perceived fairness could be effective ways to reduce their feelings of humiliation and coercion during the hospital stay, and consequently improve their overall satisfaction with treatment. Interventions aimed at reducing experienced coercion and its negative effects should take these stage-specific elements into account and proposed tailored strategies to address them.

### Electronic supplementary material

Below is the link to the electronic supplementary material.

Supplementary Material 1

## Data Availability

The datasets used and analysed during the current study are available from the corresponding author on reasonable request.

## Abbreviations

AES: MacArthur Admission Experience Survey
CES: Coercion Experience Scale
CES-H: Coercion Experience Scale - Humiliation/coercion sub-scale
CES-PAE: Coercion Experience Scale - Physical adverse effects sub-scale
CES-IS: Coercion Experience Scale - Interpersonal separation sub-scale e
CES-NEI: Coercion Experience Scale - Negative environmental influences sub-scale
CES-F: Coercion Experience Scale - Fear sub-scale
CDIS-P: Clinical Decision-making Involvement and Satisfaction scale
CDIS-S: Clinical Decision-making Involvement and Satisfaction scale - Satisfaction sub-scale
CDIS-I: Clinical Decision-making Involvement and Satisfaction scale - Implication sub-scale
ANQ: Swiss National Association for Quality development in hospitals and clinics questionnaire
