# Supplementary material for: Pathways to experienced coercion during psychiatric admission: a network analysis
Source: BMC Psychiatry. 2024 Aug 2;24:546. doi: 10.1186/s12888-024-05968-w (PMC11295432; doi:10.1186/s12888-024-05968-w)
Supplement: Supplementary file 1 — Supplementary Material 1 [file 12888_2024_5968_MOESM1_ESM.pdf]

Table 1s. Descriptive statistics of the variables included in the network analysis			
	Mean (SD)	Median (IQR)	Skewness
AES total score	4.58 (4.10)	3.00 (6.68)	0.775
CES Humiliation/coercion score	21.92 (17.99)	17.00 (27.50)	0.642
CES Physical adverse effects score	1.46 (2.58)	0.00 (2.00)	1.98
CES Interpersonal separation score	1.95 (2.38)	1.00 (4.00)	1.02
CES Negative environmental influences score	3.29 (3.41)	2.00 (5.00)	1.13
CES Fear score	0.99 (1.86)	0.00 (1.00)	2.07
Index of Fairness	15.56 (4.80)	17.00 (7.00)	-0.940
Index of Effectiveness	13.05 (4.53)	14.00 (6.00)	-0.430
Informal coercion	0.45 (0.76)	0.00 (1.00)	1.86
Formal coercion	0.52 (0.93)	0.00 (1.00)	2.12
CDIS Implication score	1.95 (1.44)	2.00 (2.00)	0.01
CDIS Satisfaction score	3.77 (1.18)	4.17 (1.67)	-0.89
ANQ score	21.31 (5.61)	22.00 (7.00)	-0.51

Note. SD standard deviation; IQR interquartile range; AES MacArthur Admission Experience Survey; CES H Coercion Experience Scale: Humiliation/coercion sub-scale; CES PAE Coercion Experience Scale: Physical adverse effects sub-scale; CES IS Coercion Experience Scale: Interpersonal separation sub-scale; CES NE Coercion Experience Scale: Negative environmental influences sub-scale; CES F Coercion Experience Scale: Fear sub-scale; CDIS I Clinical Decision-making Involvement and Satisfaction scale: Implication sub-scale; CDIS S Clinical Decision-making Involvement and Satisfaction scale: Satisfaction sub-scale ANQ Satisfaction questionnaire developed by the Swiss National Association for Quality development in hospitals and clinics.

Table 2s. Socio-demographic and clinical characteristics of the study sample (N = 225)		
Characteristics	n	%
Involuntary hospitalisation	71	31.6
Gender, female	120	53.3
Age (mean±SD)	39.2±13.7	
Swiss Nationality	165	73.3
Marital status, single	133	59.1
Main Diagnosis		
Mental and behavioural disorders due to psychoactive substance use (F10)	18	8.3
Mental and behavioural disorders due to psychoactive substance use (F11-F19)	8	3.7
Schizophrenia (F20-F29)	57	26.1
Mood affective disorders (F30-F31)	27	12.4
Mood affective disorders (F32-F39)	55	25.2
Neurotic, stress-related and somatoform disorders (F40-F48)	18	8.3
Personality disorders (F60-F69)	34	15.6
Psychological development disorders (F80-F89)	1	0.5
No diagnostic information available	7	3.1

Table 3s Weighted adjacency matrix

Variable	AES	CES H	CES PAE	CES IS	CES NEI	CES F	Index of Fairness	Index of Effectiveness	Informal coercion	Formal coercion	CDIS I	CDIS S	ANQ
AES	0.000	0.191	0.024	0.000	0.000	0.000	0.000	0.000	0.000	0.093	-0.394	-0.240	0.000
CES H	0.191	0.000	0.187	0.235	0.158	0.052	-0.162	0.000	0.083	0.130	0.000	-0.190	-0.093
CES PAE	0.024	0.187	0.000	0.058	0.148	0.000	0.000	-0.044	0.053	0.000	0.000	-0.005	0.000
CES IS	0.000	0.235	0.058	0.000	0.157	0.000	-0.071	0.000	0.018	0.000	0.000	-0.020	-0.158
CES NE	0.000	0.158	0.148	0.157	0.000	0.148	0.000	0.000	0.047	0.000	0.000	0.000	0.000
CES F	0.000	0.052	0.000	0.000	0.148	0.000	0.000	0.000	0.100	0.000	0.000	0.000	0.000
Index of Fairness	0.000	-0.162	0.000	-0.071	0.000	0.000	0.000	0.167	-0.041	0.000	0.021	0.111	0.323
Index of Effectiveness	0.000	0.000	-0.044	0.000	0.000	0.000	0.167	0.000	0.000	0.000	0.000	0.277	0.133
Informal coercion	0.000	0.083	0.053	0.018	0.047	0.100	-0.041	0.000	0.000	0.137	-0.081	0.000	0.000
Formal coercion	0.093	0.130	0.000	0.000	0.000	0.000	0.000	0.000	0.137	0.000	-0.076	-0.067	0.000
CDIS I	-0.394	0.000	0.000	0.000	0.000	0.000	0.021	0.000	-0.081	-0.076	0.000	0.064	0.000
CDIS S	-0.240	-0.190	-0.005	-0.020	0.000	0.000	0.111	0.277	0.000	-0.067	0.064	0.000	0.085
ANQ	0.000	-0.093	0.000	-0.158	0.000	0.000	0.323	0.133	0.000	0.000	0.000	0.085	0.000

Note. AES MacArthur Admission Experience Survey; CES H Coercion Experience Scale: Humiliation/coercion sub-scale; CES PAE Coercion Experience Scale: Physical adverse effects sub-scale; CES IS Coercion Experience Scale: Interpersonal separation sub-scale; CES NE Coercion Experience Scale: Negative environmental influences sub-scale; CES F Coercion Experience Scale: Fear sub-scale; CDIS I Clinical Decision-making Involvement and Satisfaction scale: Implication sub-scale; CDIS S Clinical Decision-making Involvement and Satisfaction scale: Satisfaction sub-scale ANQ Satisfaction questionnaire developed by the Swiss National Association for Quality development in hospitals and clinics.

Table 4s Centrality indices

Variable	Raw score		Standardized score	
	Strength	Expected influence	Strength	Expected influence
AES	0.943	-0.327	0.661	-1.713
CES H	1.479	0.590	2.446	1.060
CES PAE	0.519	0.421	-0.754	0.549
CES IS	0.716	0.219	-0.095	-0.063
CES NEI	0.658	0.658	-0.290	1.266
CES F	0.299	0.299	-1.485	0.181
Index of Fairness	0.896	0.349	0.503	0.330
Index of Effectiveness	0.621	0.533	-0.413	0.888
Informal coercion	0.559	0.316	-0.618	0.233
Formal coercion	0.504	0.216	-0.804	-0.072
CDIS I	0.637	-0.466	-0.359	-2.135
CDIS S	1.060	0.015	1.050	-0.679
ANQ	0.792	0.291	0.158	0.156

Note. AES MacArthur Admission Experience Survey; CES H Coercion Experience Scale: Humiliation/coercion sub-scale; CES PAE Coercion Experience Scale: Physical adverse effects sub-scale; CES IS Coercion Experience Scale: Interpersonal separation sub-scale; CES NE Coercion Experience Scale: Negative environmental influences sub-scale; CES F Coercion Experience Scale: Fear sub-scale; CDIS I Clinical Decision-making Involvement and Satisfaction scale: Implication sub-scale; CDIS S Clinical Decision-making Involvement and Satisfaction scale: Satisfaction sub-scale ANQ Satisfaction questionnaire developed by the Swiss National Association for Quality development in hospitals and clinics.

Figure 1s. Accuracy of edge-weight estimates and the associated 95% confidence intervals

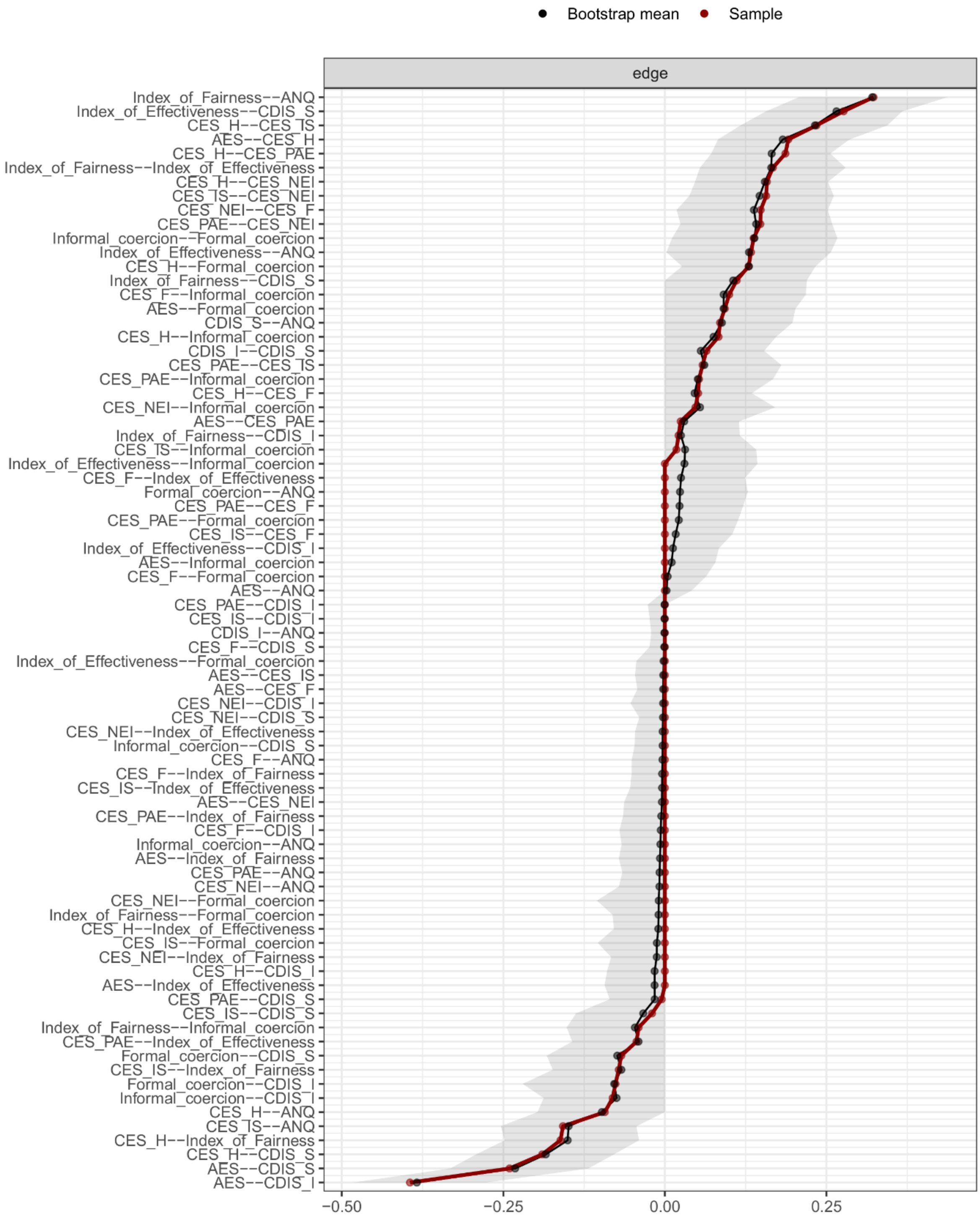

Note. AES MacArthur Admission Experience Survey; CES H Coercion Experience Scale: Humiliation/coercion sub-scale; CES PAE Coercion Experience Scale: Physical adverse effects sub-scale; CES IS Coercion Experience Scale: Interpersonal separation sub-scale; CES NE Coercion Experience Scale: Negative environmental influences sub-scale; CES F Coercion Experience Scale: Fear sub-scale; CDIS I Clinical Decision-making Involvement and Satisfaction scale: Implication sub-scale; CDIS S Clinical Decision-making Involvement and Satisfaction scale: Satisfaction sub-scale; ANQ Satisfaction questionnaire developed by the Swiss National Association for Quality development in hospitals and clinics.

Figure 2s. Bootstrapped difference test for edge-weights

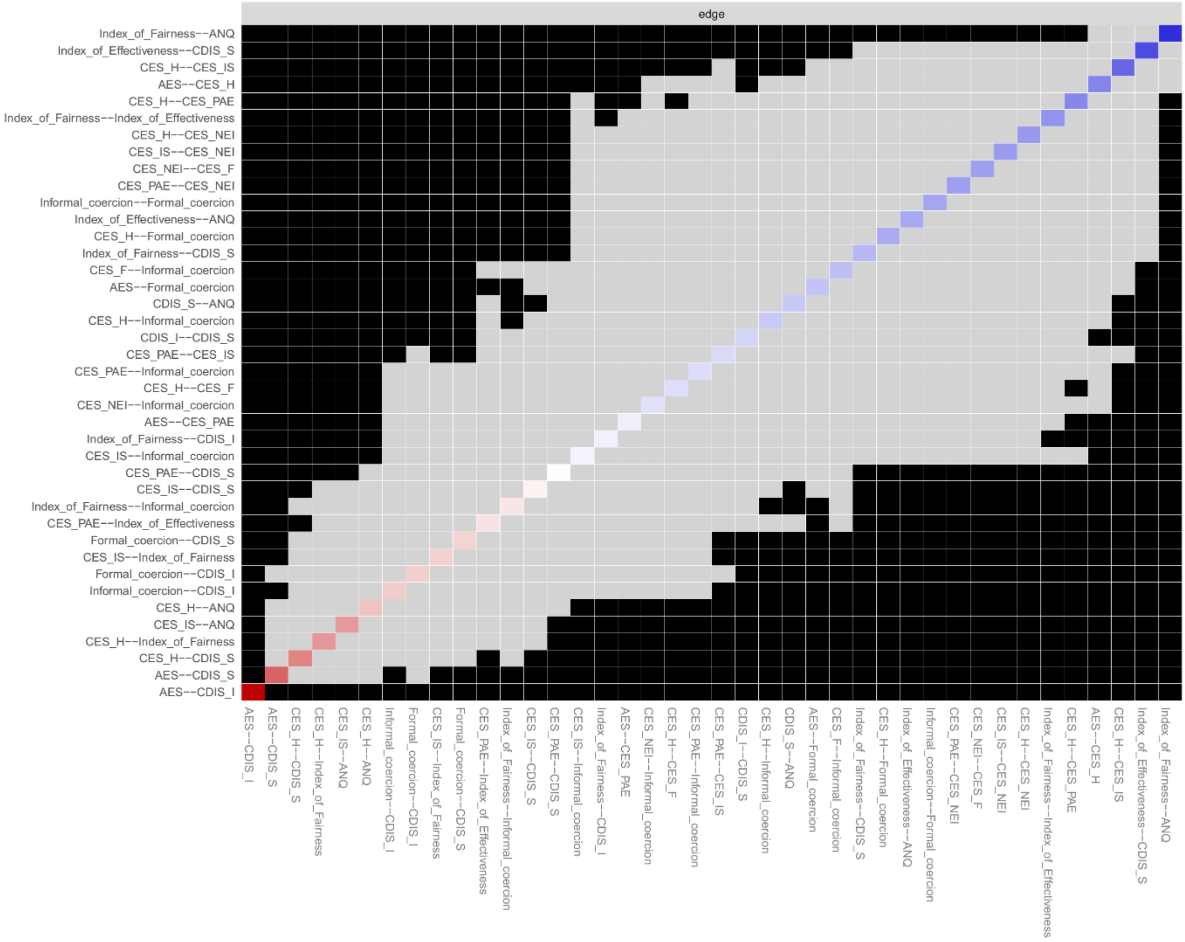

Note. AES MacArthur Admission Experience Survey; CES H Coercion Experience Scale: Humiliation/coercion sub-scale; CES PAE Coercion Experience Scale: Physical adverse effects sub-scale; CES IS Coercion Experience Scale: Interpersonal separation sub-scale; CES NE Coercion Experience Scale: Negative environmental influences sub-scale; CES F Coercion Experience Scale: Fear sub-scale; CDIS I Clinical Decision-making Involvement and Satisfaction scale: Implication sub-scale; CDIS S Clinical Decision-making Involvement and Satisfaction scale: Satisfaction sub-scale; ANQ Satisfaction questionnaire developed by the Swiss National Association for Quality development in hospitals and clinics.

Figure 3s. Stability of centrality indices by case dropping subset bootstrap

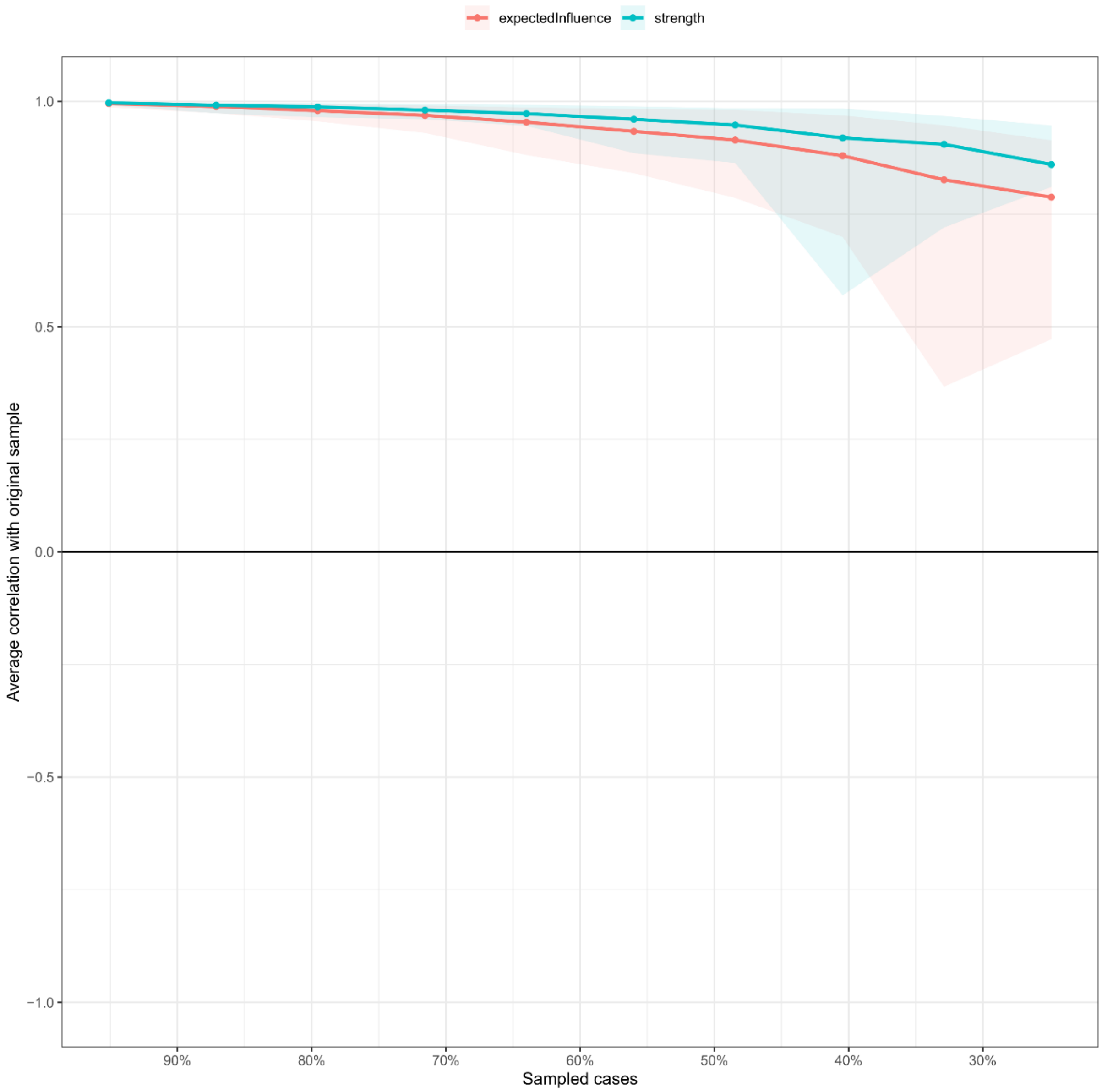

Figure 4s. Bootstrapped difference test for strength centrality estimates

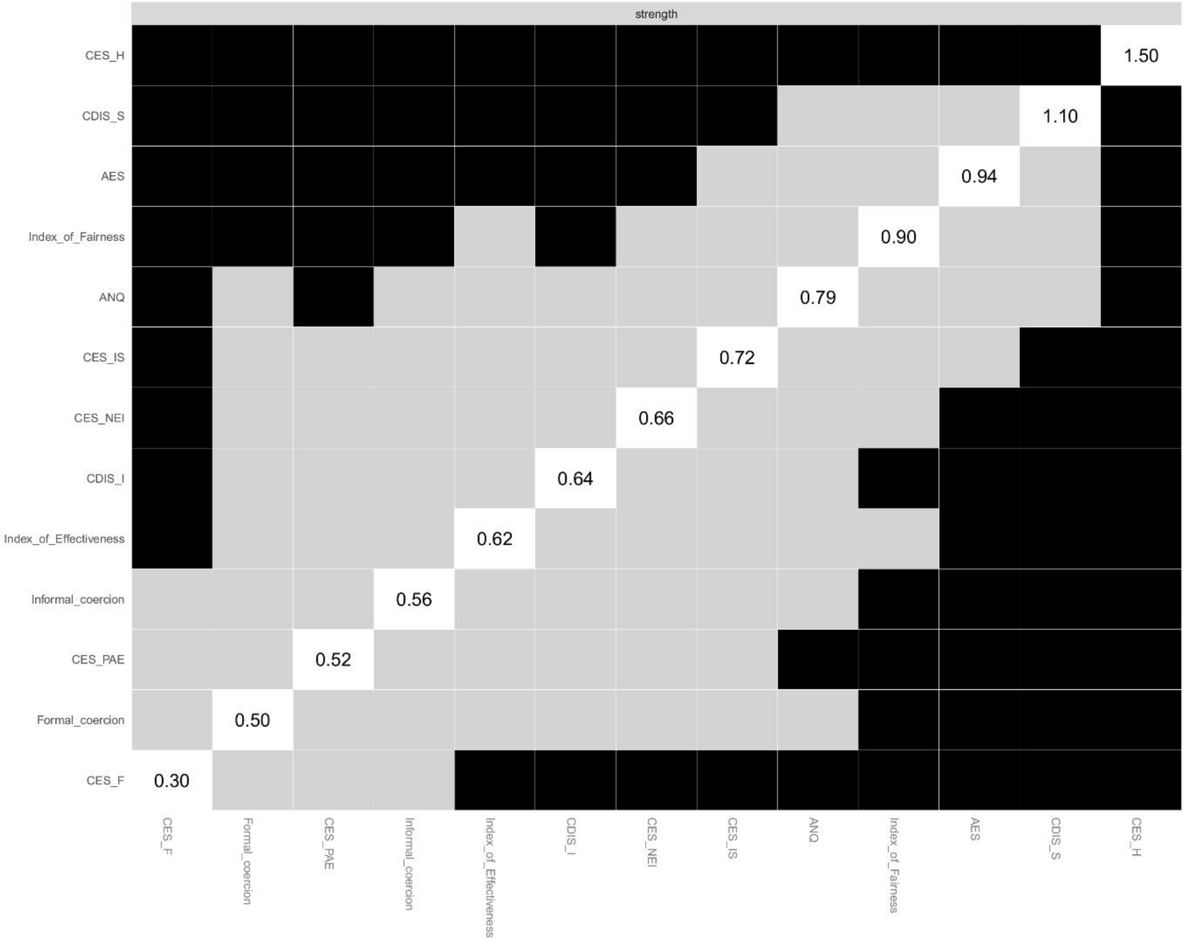

Note. AES MacArthur Admission Experience Survey; CES H Coercion Experience Scale: Humiliation/coercion sub-scale; CES PAE Coercion Experience Scale: Physical adverse effects sub-scale; CES IS Coercion Experience Scale: Interpersonal separation sub-scale; CES NE Coercion Experience Scale: Negative environmental influences sub-scale; CES F Coercion Experience Scale: Fear sub-scale; CDIS I Clinical Decision-making Involvement and Satisfaction scale: Implication sub-scale; CDIS S Clinical Decision-making Involvement and Satisfaction scale: Satisfaction sub-scale; ANQ Satisfaction questionnaire developed by the Swiss National Association for Quality development in hospitals and clinics.

Figure 5s. Bootstrapped difference test for expected influence estimates

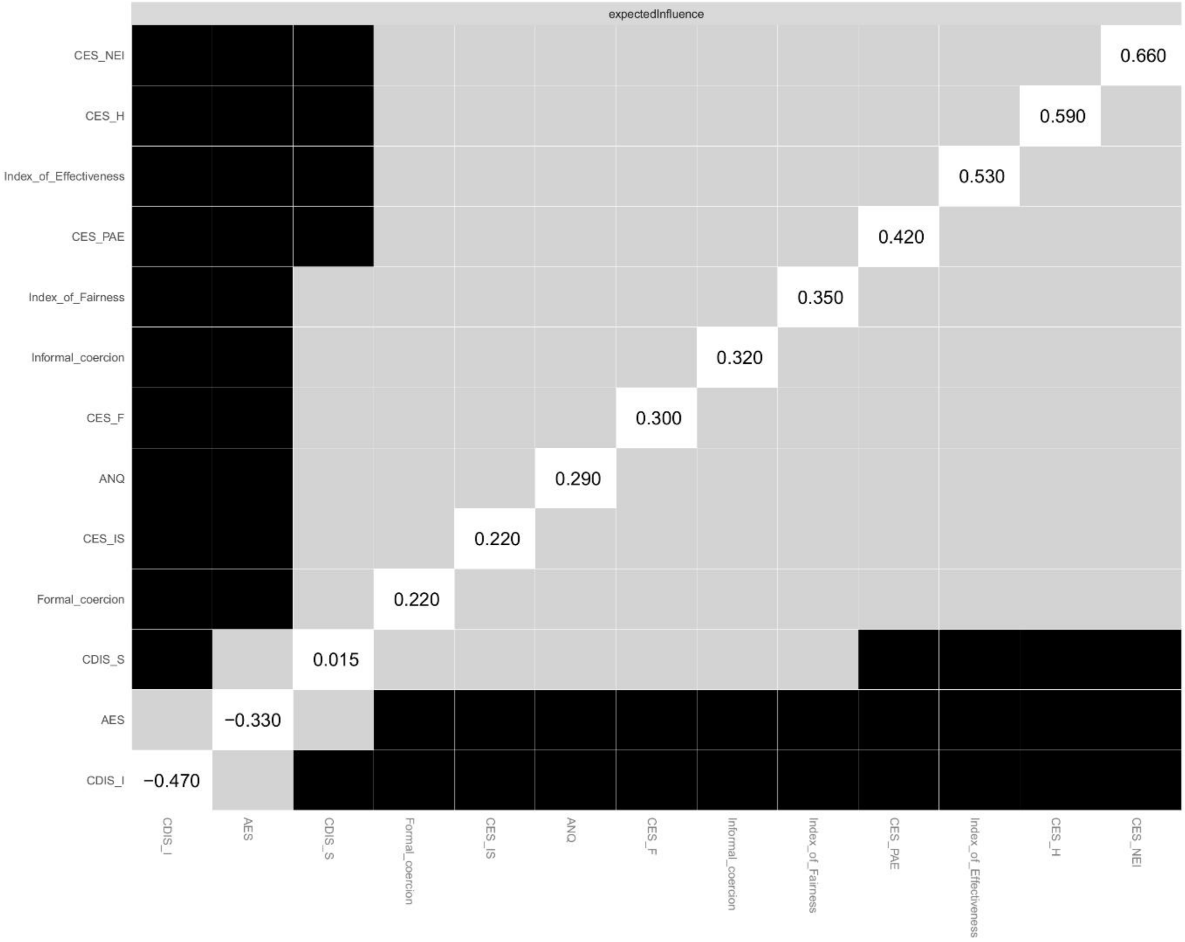

Note. AES MacArthur Admission Experience Survey; CES H Coercion Experience Scale: Humiliation/coercion sub-scale; CES PAE Coercion Experience Scale: Physical adverse effects sub-scale; CES IS Coercion Experience Scale: Interpersonal separation sub-scale; CES NE Coercion Experience Scale: Negative environmental influences sub-scale; CES F Coercion Experience Scale: Fear sub-scale; CDIS I Clinical Decision-making Involvement and Satisfaction scale: Implication sub-scale; CDIS S Clinical Decision-making Involvement and Satisfaction scale: Satisfaction sub-scale; ANQ Satisfaction questionnaire developed by the Swiss National Association for Quality development in hospitals and clinics.
